# Characterization of *Erwinia gerundensis* A4, an Almond-Derived Plant Growth-Promoting Endophyte

**DOI:** 10.3389/fmicb.2021.687971

**Published:** 2021-08-25

**Authors:** J. Paola Saldierna Guzmán, Mariana Reyes-Prieto, Stephen C. Hart

**Affiliations:** ^1^Quantitative and Systems Biology, University of California, Merced, Merced, CA, United States; ^2^Sierra Nevada Research Institute, University of California, Merced, Merced, CA, United States; ^3^Evolutionary Systems Biology of Symbionts, Institute for Integrative Systems Biology (I^2^SysBio), University of Valencia and Spanish Research Council (CSIC), Valencia, Spain; ^4^Sequencing and Bioinformatics Service of the Foundation for the Promotion of Health and Biomedical Research of the Valencia Region (FISABIO), Valencia, Spain; ^5^Department of Life and Environmental Sciences, University of California, Merced, Merced, CA, United States

**Keywords:** *Erwinia gerundensis*, endophyte, *Prunus dulcis*, plant growth promotion, spermidine, siderophore, phosphate solubilization

## Abstract

The rapidly increasing global population and anthropogenic climate change have created intense pressure on agricultural systems to produce increasingly more food under steadily challenging environmental conditions. Simultaneously, industrial agriculture is negatively affecting natural and agricultural ecosystems because of intensive irrigation and fertilization to fully utilize the potential of high-yielding cultivars. Growth-promoting microbes that increase stress tolerance and crop yield could be a useful tool for helping mitigate these problems. We investigated if commercially grown almonds might be a resource for plant colonizing bacteria with growth promotional traits that could be used to foster more productive and sustainable agricultural ecosystems. We isolated an endophytic bacterium from almond leaves that promotes growth of the model plant *Arabidopsis thaliana*. Genome sequencing revealed a novel *Erwinia gerundensis* strain (A4) that exhibits the ability to increase access to plant nutrients and to produce the stress-mitigating polyamine spermidine. Because *E. gerundensis* is known to be able to colonize diverse plant species including cereals and fruit trees, A4 may have the potential to be applied to a wide variety of crop systems.

## Introduction

Climate change and the growing world population create increasing pressure for agricultural systems to produce sufficient food (United Nations, 2019). To compensate for this demand, modern agriculture uses high-yielding cultivars and applies abundant irrigation water, fertilizers, and pesticides (Martinho, 2020). Irrigation water is not only a limited resource, but its excessive use can also negatively impact yield and the environment. For example, intensive irrigation can lead to loss of fertile soils and increased soil salinity (Dale and Polasky, 2007). Additionally, these surface runoffs can contaminate surface water as well as groundwater (Dale and Polasky, 2007; Martinho, 2020).

Almonds are an example of a high demanding agricultural product (Zhang et al., 2019). They are important for diverse culinary cultures around the world, and their consumption can improve cholesterol and blood sugar levels (Li et al., 2011; Ortiz et al., 2012; Choudhury et al., 2014; Berryman et al., 2015). These positive attributes among others have led to a high market demand that requires large fertilizer inputs and ample amounts of water usage to fully utilize the yield potential of almond trees (Zhang et al., 2019). For example, almond tree irrigation requirements are high, with estimates of 12 L of water to produce a single almond kernel (Fulton et al., 2019). To mitigate the direct and indirect negative impacts of modern agriculture on natural and agroecosystems, more sustainable strategies are required. Utilizing bacteria could be a possible approach to counter the negative impacts of modern agriculture (Compant et al., 2010). Diverse bacteria living in association with plants are able to increase the stress tolerance and nutrient availability of their host and, therefore, could reduce the need for irrigation and fertilizer inputs (Sturz et al., 2000; Lodewyckx et al., 2002; Ryu et al., 2004; Chen et al., 2010; Yandigeri et al., 2012; Timmusk et al., 2014; Soares et al., 2016; Verma et al., 2018).

In many soil types, negatively charged forms of phosphorus (P) are attached to cations, such as iron, aluminum, and calcium (Hinsinger, 2001). Microbes are crucial for making this P availability for plants. For example, bacterial enzymes can increase in the proton concentration of the soil via the production of organic acids, and this pH change solubilizes inorganic P (Kafle et al., 2019). One example is the bacterial enzyme glucose dehydrogenase that is encoded by the *gcd* gene and which uses glucose for the synthesis of the organic acid gluconic acid (Goldstein, 1995; Liang et al., 2020). This enzyme requires the bacterial redox active cofactor pyrroloquinoline quinone (PQQ), which is produced by six Pqq proteins (Anthony, 2001; Shen et al., 2012). The presence of *gcd* and the *pqq* cluster indicates the bacterial strains’ ability to lower the pH of alkaline soils in order to increase availability of phosphorous and iron. Furthermore, bacteria are able to produce siderophores, low-molecular-mass molecules with a high affinity for iron (Fe; Richardson et al., 1999). For example, enterobactin has an exceptionally high affinity for Fe^3+^ (K_a_ = 10^52^) and is synthesized by several enzymes encoded by *ent* genes (Avdeef et al., 1978; Raymond et al., 2003; McRose et al., 2018). Several studies have shown that plants are able to access Fe by the uptake of microbial siderophores (Bar-Ness et al., 1992; Vansuyt et al., 2007; Jin et al., 2010; Shirley et al., 2011).

Plants synthesize low-molecular-mass linear polyamines, like spermidine, that are essential for plant growth (Kusano et al., 2008). Supplemental spermidine, provided either by overexpression of spermidine synthetase or exogenous application, enhances plant defense responses and increases tolerance to diverse abiotic stresses, like salinity and drought (Yoda et al., 2003; Kasukabe et al., 2004; Moschou et al., 2009). Overall, these findings suggest that increasing spermidine could improve plant tolerance to diverse environmental stresses. Bacteria are a potential source for providing supplemental spermidine to plants (Xie et al., 2014). They can synthesize spermidine from the two amino acids, methionine and arginine, catalyzed by enzymes encoded by the *metK* and *spe* genes (Tabor and Tabor, 1985; Shah and Swiatlo, 2008; Guerra et al., 2018).

To identify potential resources for a more sustainable agriculture, the primary goal of this study was to isolate and characterize a growth-promoting bacterial endophyte from almond leaves that carries genes to improve nutrient availability and plant stress tolerance. We successfully isolated a growth-promoting bacterium, and whole genome sequencing analysis identified this bacterium as novel strain of *Erwinia gerundensis*. This bacterial species has been found in diverse agricultural ecosystems around the globe. This suggests that the bacterial strain described here has a potential use not only in almonds but also for a wide variety of other crops.

## Materials and Methods

### Sample Collection and Leaf Tissue Sterilization

**FIGURE 1:**
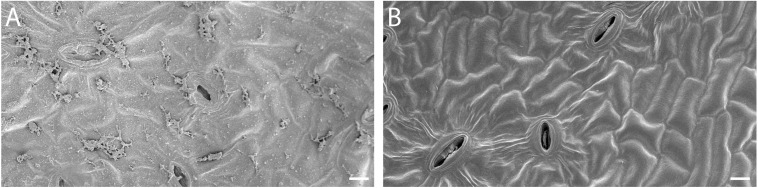
Removal of surface microbes from almond leaves **(A)** Before and **(B)** after surface sterilization. Scale bar = 10 μm.

Leaves of visually healthy almond trees growing in an orchard located in Modesto, California (37°42′21.8″N, 120°56′55.1″W) were collected aseptically in July 2019. Leaves were placed in sterile bags and immediately stored at 4°C in a portable cooler and then transported to the laboratory at the University of California, Merced. Within 3 h after collection, leaves were surface sterilized using 8.25% sodium hypochlorite and afterward rinsed twice with sterile water. Removal of epiphytic microbes was confirmed using scanning electron microscopy (SEM; Figure 1). Surface sterilization and SEM procedures were previously described for cottonwood leaves (Saldierna Guzmán et al., 2020).

### Enrichment and Isolation of Endophytic Bacterial Isolates

Immediately after removing epiphytic microbes, 20 g of surface sterilized leaves were blended using bacterial cell extraction buffer (50 mM Tris–HCl [pH7.5], 1% Triton X-100, 2 mM β-mercaptoethanol) for 3 min to enrich for endophytic bacterial cells (Ikeda et al., 2009). We found that filtering through Miracloth and centrifugation at 500 × *g* was sufficient to remove plant cell debris. Therefore, we excluded the Nycodenz density gradient centrifugation described in Ikeda et al. (2009). The enriched endophytic bacterial cells were resuspended using 1x phosphate-buffered saline (PBS). Ten microliters of this suspension was plated on Lysogeny Broth (LB) agar media (Bertani, 1951) or on Norris Glucose Nitrogen Free Media (HIMEDIA, Mumbai, Maharashtra, India), and then incubated 5–7 days at 28°C. Subsequently, the obtained colonies were repeatedly streaked and incubated on LB agar media (Bertani, 1951) to obtain pure isolates. A total of 100 bacterial isolates were obtained from surface-sterilized almond leaves. Of these bacteria, 22 isolates were randomly selected and tested for their plant growth-promoting abilities (see below under “Plant inoculation”). Only one of these 22 isolates, named A4, was chosen for its plant growth-promoting ability.

In order to identify the isolates, 16S rRNA gene amplification of each sample was conducted using universal primers 27f and 1492r (Lane, 1991). The 50-μl PCR contained 1x complete reaction buffer, 200 μM dNTPs, 0.5 μM forward primer, 0.5 μM reverse primer, 10 ng of template DNA, and 1.25 U DFS-Taq DNA polymerase (BIORON GmbH, Römerberg, Rheinland-Pfalz, Germany). Cycling conditions were 95°C for 2 min, followed by 25 cycles of 95°C for 30 s, 55°C for 30 s, 72°C for 2 min, with a final extension of 72°C for 10 min. All samples were purified from 1.5% agarose gels using the Zymoclean Gel DNA Recovery Kit (Zymo research, Irvine, CA, United States) following the recommended procedure of the manufacturer. Subsequently, amplicons were sent for Sanger sequencing at Eton Bioscience (San Diego, CA, United States). The Sanger sequencing results have been uploaded to the NCBI database and can be found under the GenBank accession numbers MZ227353–MZ227374.

### Competent Cells and Transformation of Endophytic A4 Bacterial Strain

In order to transform the strain A4 with a plasmid that carries maker genes for visualization of A4 in plant tissues, competent cells were generated. For this purpose, A4 cells were grown overnight at 28°C until they reached an optical density (OD_600_) of 0.5. They were cooled in an ice bath and washed with sterile, cold water four times after centrifugation steps at 4,000 × *g*. Afterward, cells were resuspended in 10% glycerol, aliquoted, and snap frozen in liquid nitrogen. Electroporation was used for transformation (Cadoret et al., 2014) of A4 with the pRU1156 plasmid, Addgene catalog 14473 (Karunakaran et al., 2005).

### Western Blotting

Transformed and untransformed A4 strains were grown on LB liquid media overnight at 28°C. Bacterial suspensions were normalized by measuring OD_600_ of 0.1, and equal amounts of cells were boiled in sample loading buffer (Sigma-Aldrich, Darmstadt, Germany) and separated in SDS-PAGEs. For immunoblotting, anti-GFP (1:5,000, Roche, 11814460001, Darmstadt, Germany) was used to detect free GFP (Bürger et al., 2017).

### Plant Inoculation

*Arabidopsis thaliana* (ecotype Columbia-0) seeds were surface sterilized by hydrochloric fumigation for 3 h and stratified for 3–5 days at 4°C (Lindsey et al., 2017). Subsequently, the sterile seeds were grown *in vitro* on half-strength Linsmaier and Skoog (LS) medium at pH 5.7 with 7% plant agar. Seedlings were grown on vertically oriented plates in a growth chamber (Percival, Iowa, United States) with continuous white light (100 μmol m^– 2^ s^– 1^) at 21°C (Qiu et al., 2019). Prior to inoculation, the 22 selected endophytic isolates were grown for 24 h in LB media with 50 μg tetracycline ml^– 1^ and 100 μg ampicillin ml^– 1^ at 28°C. The bacterial cells were centrifuged at 3,500 × *g*, resuspended in infiltration buffer (10 mM MgSO_4_, 10 mM MES-KOH pH 5.5), and adjusted to an OD_600_ of 0.1 in the same buffer. Subsequently, the roots of 7-days old seedlings were inoculated with 2–5 μl of bacterial inoculum per root, being careful not to contaminate shoot tissues, following the protocol for *Salmonella enterica* and *Escherichia coli* O157:H7 (Cooley et al., 2003). Infiltration buffer without bacteria served as the negative control (mock treatment).

### Plant Growth Promotion Assay

The effect of the 22 bacterial isolates on *Arabidopsis* growth was evaluated 2 weeks postinoculation. Three independent experiments were performed with 38–41 seedlings per treatment in each experiment. Fresh mass of seedlings was measured and compared to mock (infiltration buffer)-treated plants, which served as negative control. Statistical analysis was performed by one-way ANOVA with Tukey’s test using GraphPad Prism 7 (GraphPad Software, San Diego California, United States). A difference was considered statistically significant using an *a priori* determined α level of 0.05.

### GUS and Green Fluorescent Protein (GFP)

In order to test colonization of the endophytic strain A4, plant tissues were histochemically stained with X-Gluc (GUS) 7 days after inoculation of *A. thaliana* seedlings and observed with a stereomicroscope (Leica MZ16, Leica, Germany; Willige et al., 2011). Moreover, seedlings were screened for GFP expression using a Zeiss LSM 710 fluorescence microscope (Carl Zeiss, Germany).

### Genome Sequencing

The bacterial strain A4 was sent to Novogene Biotech (Beijing, China) for DNA extraction, library preparation, and whole genome sequencing and assembly using PacBio Single Molecule, Real-Time (SMRT) Sequencing (Eid et al., 2009). Falcon software (Chin et al., 2016) was used for genome assembly, and BUSCO for the assessment of the genome assembly, gene set, and transcriptome completeness (Simão et al., 2015). Prokka software was used for genome annotation (Seemann, 2014), and functional annotation was done by aligning the sequence with sequences previously deposited in diverse protein databases, including the National Center for Biotechnology Information (NCBI) non-redundant protein (Nr) database, UniProt/Swiss-Prot, Kyoto Encyclopedia of Genes and Genomes (KEGG), and Cluster of Orthologous Groups of proteins (COG).

### Bioinformatics

The phylogenetic tree was constructed with SpeciesTreeBuilder v 0.1.0 from the Kbase platform (Arkin et al., 2018). The Artemis Comparison Tool (ACT; Carver et al., 2005) was used to compare our bacterium genome with an already sequenced genome. To display circular comparisons between both genomes and plasmids, the Blast Ring Image Generator (BRIGS) was used (Alikhan et al., 2011). Finally, a nucleotide identity analysis between strain A4 and a publicly available, closely related bacterial genome was performed with the ANI calculator from EZbiocloud (Yoon et al., 2017).

### Phosphate Solubilization

Strain A4 was tested for its ability to solubilize phosphate using NBRIP medium (Nautiyal, 1999). Prior to inoculation, strain A4 was grown at 28°C until they reached an OD_600_ of 0.5. An aliquot of 10 μl was spotted on plates containing National Botanical Research Institute’s Phosphate growth medium (NBRIP; Nautiyal, 1999) and incubated at 28°C for 7 days.

### Siderophore

Siderophore production was determined by chrome azurol S (CAS) agar plates. The medium was prepared according to the method described by Schwyn and Neilands (1987). Strain A4 was grown overnight in LB liquid medium at 28°C. A4 bacterial suspension was normalized by adjusting to an OD_600_ of 0.5 with LB medium. Subsequently, 10 μl of the suspension was spotted on CAS agar plates and incubated for 7 days at 28°C.

## Results

### Isolation, Characterization, and Effect of A4 Endophyte on Plant Growth

**FIGURE 2:**
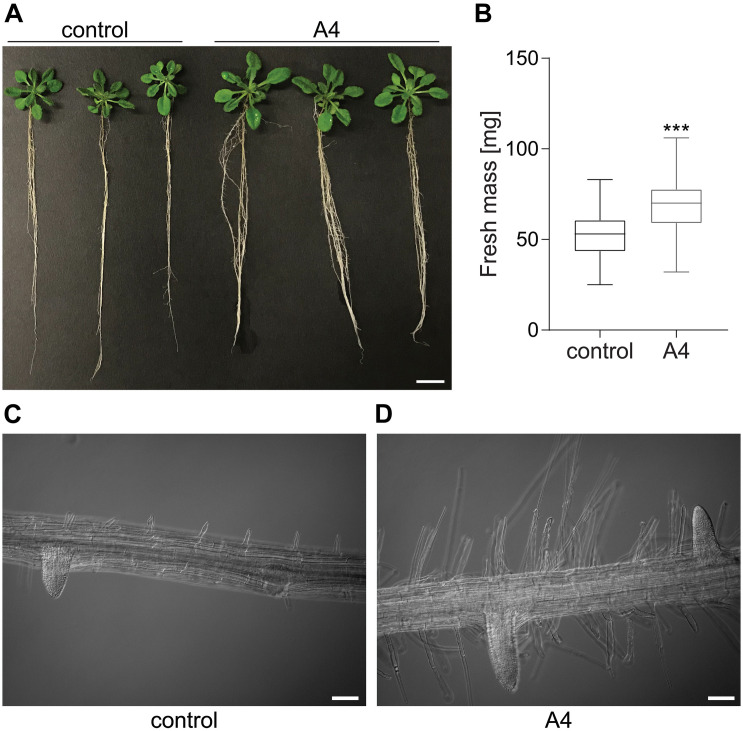
Effect of endophytic strain A4 on *Arabidopsis thaliana* **(A)** Photo taken after 2 weeks postinoculation in comparison to the control. Scale bar = 1 cm. **(B)** Fresh mass of seedlings compared to the control (one-way ANOVA, Tukey’s multiple comparisons test, *** *p* ≤ 0.001), *n* = 41. **(C,D)** Differences in the root after 2 weeks of inoculation with A4 in contrast to the control. Scale bar = 0.2 mm. Control for all experiments: mock (infiltration buffer)-treated plants.

Twenty-two strains were isolated from surface-sterilized almond leaves (Figure 1). They were tested for their plant growth-promoting ability, and only the strain A4 had a positive effect on plant growth. Therefore, A4 was selected for further characterization. Sanger sequencing of the 16S rRNA gene revealed its phylogenetic relationship to the genera *Pantoea* and *Erwinia*. Three independent experiments revealed that endophyte-treated plants had about 30% higher fresh mass than control plants (*p* ≤ 0.001; Figures 2A,B). Additionally, the A4 treatment resulted in increased root hair length and abundance (Figures 2C,D). These results suggest that inoculation with an almond-derived endophyte could promote growth of *Arabidopsis* seedlings.

### Transformation of Strain A4

**FIGURE 3:**
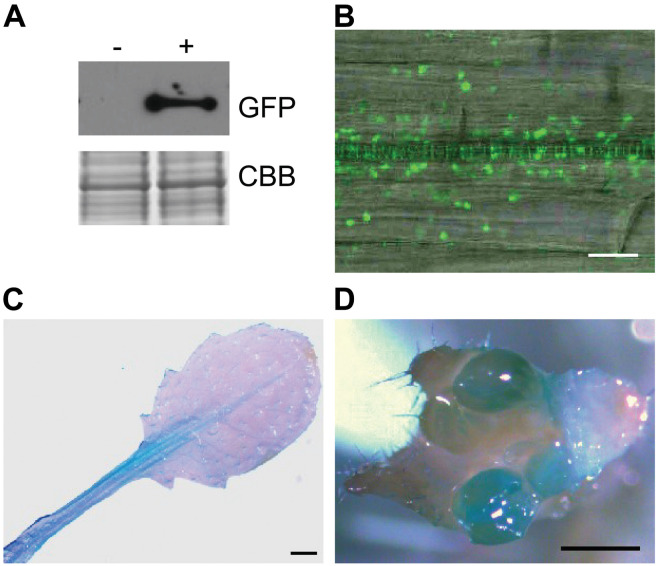
Colonization of the endophytic strain A4 **(A)** Western blot analysis of A4 transformant expressing GFP. Coomassie stain (CBB) shows equivalent protein loading. An antibody against GFP was used for visualization, and non-transformant A4 strain as a negative control. Seven days after inoculation of *Arabidopsis thaliana* seedlings, **(B)** GFP-expressing A4 inside the primary root. Scale bar = 20 μm. Histochemical GUS activity of A4 strain in **(C)** leaf tissue and **(D)** inflorescence. Scale bar = 1 mm.

To test plant colonization by A4, we transformed A4 to express the marker genes GFP and gusA in order to track the colonization of A4 within internal plant tissues. Successful transformation of A4 was confirmed by Western blotting to visualize expression of GFP encoded in the transformed plasmid (Figure 3A).

*Arabidopsis* roots were inoculated with endophytic bacteria A4 labeled with GFP to determine whether intracellular colonization was occurring by live-cell imaging. Confocal observation revealed GFP-expressing A4 colonizing internal root tissues 7 days after inoculation. Many of these bacterial cells were observed within the root vasculature (Figure 3B). Furthermore, analyses 14 days postinoculation showed that A4 had colonized shoot tissues such as leaves and flowers, as visualized by GUS staining (Figures 3C,D). These observations indicated that the root-inoculated bacteria were able to spread inside the plant and colonize above ground tissues. The mock-inoculated plants did not show any blue staining or fluorescent bacteria.

### Genomic Features of A4

The whole-genome sequence analysis of A4 was conducted in order to obtain reliable taxonomic classification and identify genes or pathways that could potentially contribute to the plant growth-promoting effects. The A4 genome consisted of a single circular chromosome of 3,858,052 base pairs (bp), with an average GC content of 55%. The A4 strain had one plasmid we named pA401, with a size of approximately 576,382 bp, and also had an average GC content of 55% like the chromosome. The chromosome contained 3,552 genes, including genes for 78 tRNAs, for 22 rRNAs, and 3,451 protein-coding sequences (CDS). Among these CDSs, 2,929 genes were classified into clusters of orthologous group (COG) families composed of 23 categories. KEGG pathway annotation resulted in the functional annotation of 3,414 genes (96%). Of these annotated genes, most were grouped into the two categories: “metabolism” and “environmental information processing” (Table 1). The A4 plasmid pA401 had 521 protein-coding sequences and 1 contig.

**TABLE 1:** Genome features of A4.

COG pathway annotation		KEGG function classification	
Function	Genes	Function	Genes
RNA processing and modification	1	**Genetic information**	
Energy production and conversion	160	Translation	81
Cell cycle control and division, chromosome partitioning	44	Transcription	4
Amino acid transport and metabolism	341	Replication and repair	54
Nucleotide transport and metabolism	92	Folding, sorting, and degradation	51
Carbohydrate transport and metabolism	307	**Cellular processes**	
Coenzyme transport and metabolism	168	Transport and catabolism	8
Lipid transport and metabolism	113	Cellular community	151
Translation, ribosomal structure, and biogenesis	239	Cell mobility	90
Transcription	247	Cell growth and death	21
Replication, recombination, and repair	127	**Metabolism**	
Cell wall/membrane/envelope biogenesis	223	Xenobiotics biodegradation and metabolism	26
Cell motility	110	Nucleotide metabolism	115
Posttranslational modification, protein turnover, chaperones	126	Metabolism of terpenoids and polyketides	32
Inorganic ion transport and metabolism	208	Metabolism of other amino acids	61
Secondary metabolites biosynthesis, transport, and catabolism	62	Metabolism of other cofactors and vitamins	157
General function prediction	253	Lipid metabolism	60
Signal transduction mechanisms	186	Glycan biosynthesis and metabolism	48
Intracellular trafficking, secretion and vesicular transport	63	Energy metabolism	124
Defense mechanisms	63	Carbohydrate metabolism	233
Extracellular structures	18	Biosynthesis of other secondary metabolites	32
Mobile: prophages, transposons	15	Amio acid metabolism	174
Unknown	183	**Environmental information processing**	
		Signal transduction	123
		Membrane transport	248

### Taxonomic Affiliation of Strain A4

**FIGURE 4:**
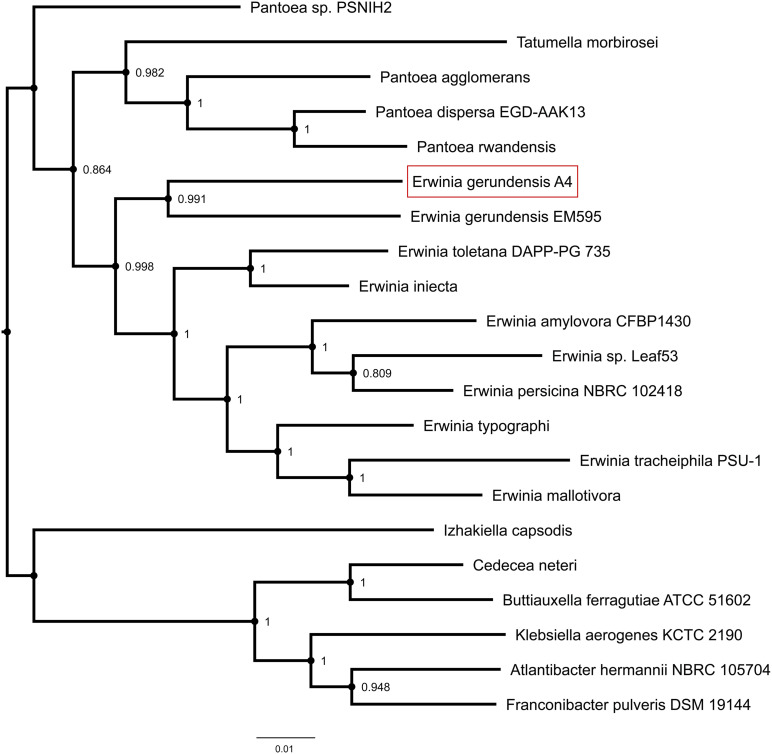
Phylogenetic tree highlighting the position of *E. gerundensis* strain A4 in comparison to publicly available genomes. Node labels represent confidence levels based on 1,000 bootstrap replicates.

**FIGURE 5:**
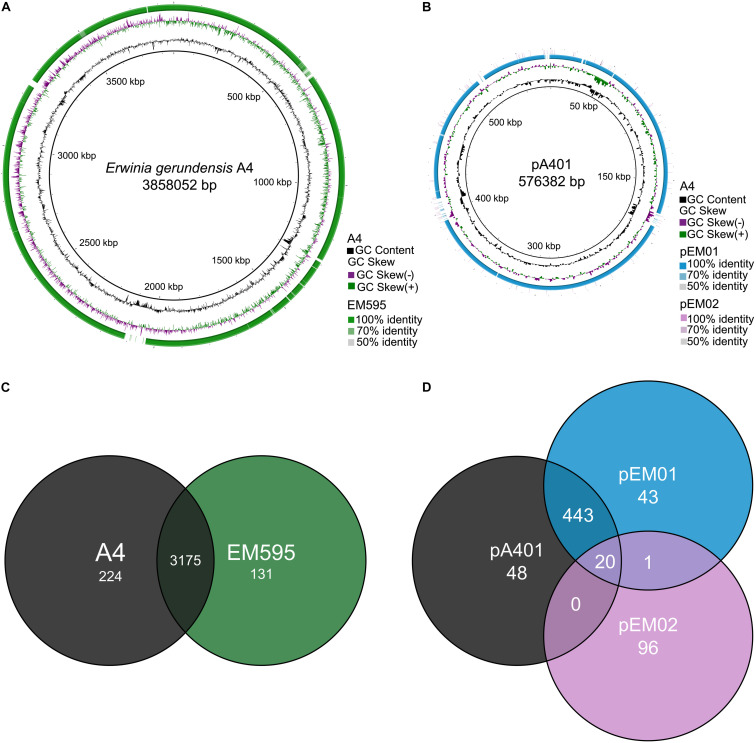
Comparison between *E. gerundensis* strain A4 and EM595. **(A,B)** Circular comparisons between both genomes and plasmids. The inner black rings depict the coordinates in scale and total size of chromosome **(A)** and plasmid **(B)** of A4. Black histograms represent GC content, while green-purple histograms show GC deviations. Orthologous sequences are displayed with the percentage of similarity. **(C,D)** Venn diagrams of chromosomes and plasmids of both strains.

We used Tree builder to search for closely related genomes and to assess the phylogenetic relationship between A4 and publicly available genomes (Figure 4 and Supplementary Table 1). This analysis revealed that A4 grouped separately from strains of *Pantoea*, but together with *Erwinia* strains and in particular very next to *E. gerundensis* strain EM595 (Rezzonico et al., 2016). Therefore, we compared chromosomes and plasmids of A4 and EM595 (Figure 5). While the chromosomes of both strains share about 90% of their genes (3175), A4 had 224 unique genes (Supplementary Table 2). Furthermore, EM595 carried two plasmids (pEM01 and pEM02), and pEM01 shared the greatest overlap with pA401 (71%, 463 genes); 20 of the common genes were also present on pEM02. Additionally, the A4 plasmid had 48 unique genes that were not present in both plasmids of the previously sequenced *Erwinia* strain EM595 (Supplementary Table 3). Taken together, these results suggest A4 represents a novel *E. gerundensis* strain.

### Plant Growth-Promoting Traits in A4

**FIGURE 6:**
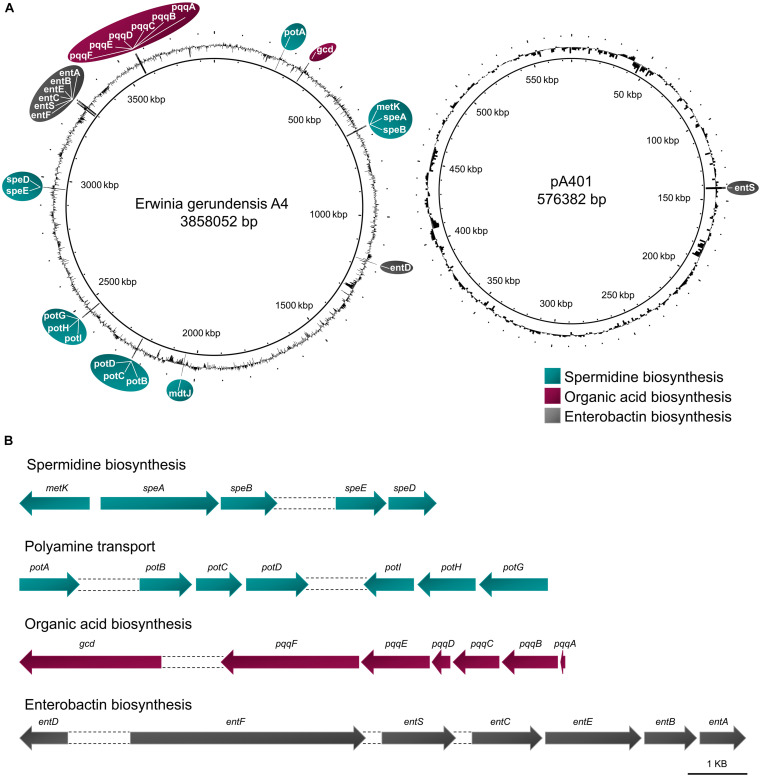
Genes encoding for plant-promoting traits in *E. gerundensis* strain A4. **(A)** Location and **(B)** cluster of genes encoding production and transport of spermidine, the phosphate-solubilizing gluconic acid, and the siderophore enterobactin.

In order to identify genes responsible for growth promotion, we searched the genome and plasmid of A4 for genes involved in nitrogen fixation, and organic acid, siderophore, and spermidine synthesis. Even though A4 was isolated on bacterial medium that is denoted as nitrogen free, we could not detect any genes encoding for nitrogenase subunits. However, we identified a *pqq* gene cluster (*pqqABCDEF*) as well as for a *gcd* gene, indicating that A4 carries all necessary enzymatic components to solubilize phosphorus by organic acid synthesis. Moreover, we found *entABCDEF* and two *entS* genes in A4, suggesting that this strain is able to produce and export the siderophore enterobactin. Furthermore, the strain carries all necessary genes (i.e., *metK* and *speABDE*) to produce the polyamine spermidine. In addition, we identified several genes involved in spermidine and putrescine transport (i.e., *potA*, *potB*, *potD*, *potG*, *potH*, and *potI*; Figure 6).

### Nutrient Acquisition by A4 Strain

**FIGURE 7:**
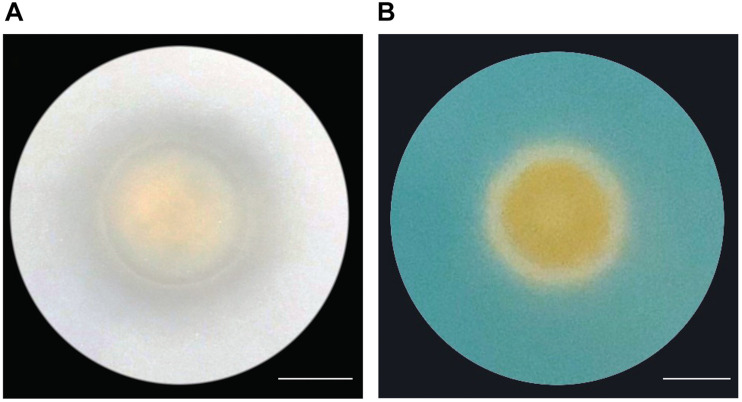
Nutrient acquisition abilities by strain A4 **(A)** Phosphate solubilization by A4. Clear zone surrounding the bacterial colony represents solubilization of Ca_3_(PO_4_)_2_. **(B)** Siderophore production in CAS medium. The color change from blue to yellow caused by siderophore production can be observed surrounding the bacterial colony. Scale bars = 10 mm.

Because the genome of A4 encodes for proteins that are involved in the production of organic acids and siderophores, we tested if A4 is able to release insoluble phosphate and synthesize iron chelators. A4 was able to produce a clear zone around the colony, suggesting phosphate solubilization activity by the production of organic acids (Figure 7A). Additionally, production and secretion of siderophores were visualized by a color change of Chrome Azurol S (CAS). In association with ferric ions, CAS appears blue, while the removal of the ferric ions by siderophores changes the color of the media from blue to yellow. Consistent with the presence of genes encoding for siderophore-synthesizing enzymes, A4 was able to cause the color change of the CAS media (Figure 7B). In contrast, a non-growth-promoting *Frigoribacterium* strain that we isolated from almond leaves was not able to solubilize phosphate or induce a yellow color of the CAS media (Supplementary Figure 1). This indicates that A4 could supply its plant host with essential nutrients such as phosphate and iron to promote growth.

## Discussion

Bacteria living in association with plants can either be harmful, neutral, or beneficial. Once a beneficial bacterium has been established on or in a plant, it can influence the physiology of the host via a variety of mechanisms. For example, bacteria can produce growth-promoting metabolites, easing the effects of biotic and abiotic stresses and providing nutrients (Bulgarelli et al., 2013). Here, we described the isolation, characterization, and sequencing of the growth-promoting *E. gerundensis* strain A4. This strain was isolated from inner leaf tissues from almonds growing in a commercial orchard. So far, only one other *E. gerundensis* strain has been sequenced, which was isolated from leaf surfaces of a pear tree (Rezzonico et al., 2016). Our sequence analysis identified A4 as a novel *E. gerundensis* strain. Furthermore, whole genome sequencing revealed that A4 exhibits the ability to produce polyamines, gluconic acid, and enterobactin.

Polyamines, like spermidine, are organic compounds produced by prokaryotes and eukaryotes that are essential for their growth and development (Kusano et al., 2008). In plants, they play a role in a variety of process like embryogenesis, root growth, flowering, fruit maturation, and retardation of senescence (Evans and Malmberg, 1989; Pandey et al., 2000). For example, in *Arabidopsis thaliana*, loss of spermidine synthase activity leads to embryo lethality (Imai et al., 2004). Other than participating in plant growth, polyamines have a protective role against several environmental stresses. Kasukabe et al. (2004) showed that overexpression of plant-derived spermidine synthase confers tolerance to freezing, salinity, drought, and osmotic stresses in *Arabidopsis thaliana*. Additionally, several studies have shown that polyamines are involved in defense responses against pathogens. The upregulated expression of polyamine synthesis genes in response to pathogenic attacks has been observed in several plant species (Fu et al., 2011; Moselhy et al., 2016). Furthermore, overexpression of spermidine synthase or exogenous application of spermidine enhances plant defense responses to viruses and bacteria (Yoda et al., 2003; Moschou et al., 2009). Altogether, these studies indicate that increasing polyamine levels minimizes the detrimental effects caused by biotic and abiotic stresses. A4 could provide spermidine and ease the damaging effects of various environmental stresses, because it carries the genes not only for spermidine synthesis but also for the export of the polyamines spermidine and putrescine.

Besides spermidine production, we identified several traits that may support nutrient acquisition of plants. For example, at a morphological level, A4 increases root growth and the elongation and density of root hairs in *Arabidopsis*. These plant growth responses increase root surface area that can enhance the access to water and the uptake of nutrients of the plant (Sukumar et al., 2013). Furthermore, the genome of A4 encodes for two enzymatic pathways involved in nutrient acquisition. Microbes developed diverse metabolic capacities to improve the bioaccessibility of recalcitrant P by the production of different organic acids (Richardson and Simpson, 2011). Bacterial PQQ-dependent glucose dehydrogenase, encoded by the *gcd* gene, catalyzes the oxidation of glucose to gluconic acid (Goldstein, 1995; Liang et al., 2020). Glucose dehydrogenase is characterized as a key enzyme necessary for phosphate solubilization in microbes. In fact, detection of the *gcd* gene in soil samples was found to be a major determinant of bioavailable P (Liang et al., 2020). Our genomic analysis of A4 identifies genes encoding for the entire enzymatic pathway for synthesis of the redox cofactor PQQ and for gluconic acid production. We found that A4 is able to solubilize Ca_3_(PO_4_)_2_, suggesting that gluconic acid is excreted by A4 to lower the pH of alkaline environments. In contrast, low soil pH leads to P fixation by iron. In acidic soils, bacteria can increase the phosphate accessibility for plants by producing siderophores that form complexes with ferric iron (Kafle et al., 2019). The genome and plasmid of A4 carry all necessary genetic information to produce and export the siderophore enterobactin. Our *in vitro* assay suggested that *ent* genes of A4 are all functional. Several studies have demonstrated that plants are able to access Fe by the uptake of microbial siderophores (Bar-Ness et al., 1992; Vansuyt et al., 2007; Jin et al., 2010; Shirley et al., 2011). This indicates that siderophores produced by growth-promoting bacteria have a dual function for the nutrient availability of the plants in acidic soils by providing phosphate as well as iron. Therefore, A4 could exhibit plant growth promotion traits not only as a leaf endophyte where it could provide spermidine to foster stress tolerance, but also as a member of the plant rhizospheres to ease access to nutrients. Furthermore, we observed floral colonization by A4. This opens the possibility that A4 might colonize reproductive tissues and may suggest that A4 might be vertically transmitted from one generation to the next.

We isolated *E. gerundensis* strain A4 from inner tissues of almond leaves and showed that A4 is able to colonize below and above ground tissues of Arabidopsis thaliana. Other *E. gerundensis* strains were found in diverse agroecosystems around the globe: Two strains were isolated from leaf surfaces of pome fruit trees in Spain, and two other strains were isolated from wheat roots in Australia (Rezzonico et al., 2009, 2016). Taken together, these results suggest that *E. gerundensis* can colonize different tissues of various plant species, including both monocots and dicots, in diverse agricultural environments. This promiscuous colonization behavior and the growth promotion traits of *E. gerundensis* A4 suggest that this strain might have the potential to improve production not only of almonds but also of a variety of other crop species around the globe.

## Data Availability Statement

The sequencing data was deposited to GenBank database under the BioProject PRJNA717909.

## Author Contributions

JSG and SH designed the research and wrote the manuscript. JSG performed the experiments. JSG and MR-P analyzed the data. All authors revised the manuscript and approved the final version.

## Conflict of Interest

The authors declare that the research was conducted in the absence of any commercial or financial relationships that could be construed as a potential conflict of interest.

## Publisher’s Note

All claims expressed in this article are solely those of the authors and do not necessarily represent those of their affiliated organizations, or those of the publisher, the editors and the reviewers. Any product that may be evaluated in this article, or claim that may be made by its manufacturer, is not guaranteed or endorsed by the publisher.

## Supplementary Material

Supplementary Figure 1 Non-growth-promoting strain *Frigoribacterium* strain isolated from almond leaves was not able to solubilize phosphate **(A)** or induce a yellow color of the CAS media **(B)**. Scale bars = 10 mm.
