# Supplementary figures and images for: Characterization of Erwinia gerundensis A4, an Almond-Derived Plant Growth-Promoting Endophyte
Source: Front Microbiol. 2021 Aug 25;12:687971. doi: 10.3389/fmicb.2021.687971 (PMC8425249; doi:10.3389/fmicb.2021.687971)

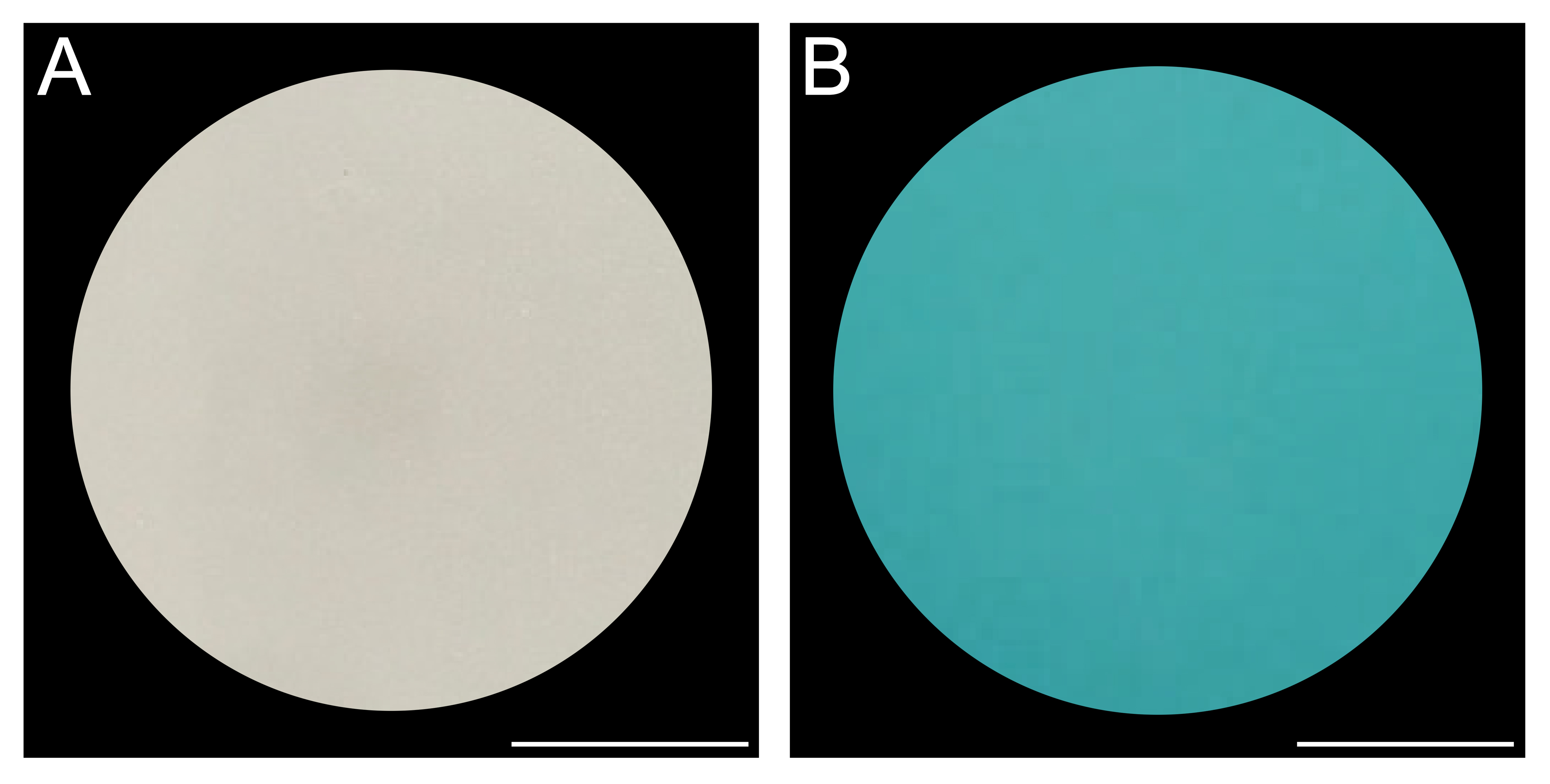

Supplement: Supplementary Figure 1 — Non-growth-promoting strain Frigoribacterium strain isolated from almond leaves was not able to solubilize phosphate (A) or induce a yellow color of the CAS media (B). Scale bars = 10 mm. [file Image_1.JPEG]
